# Physical Resilience May Offset Mortality Risks Associated With Genetic Predisposition to Shorter Survival: A Population-based Cohort Study

**DOI:** 10.1093/gerona/glaf101

**Published:** 2025-05-09

**Authors:** Lea Stark, Federico Triolo, Davide Liborio Vetrano, Debora Rizzuto, Israel Contador, Amaia Calderón-Larrañaga, Serhiy Dekhtyar

**Affiliations:** Department of Neurobiology, Care Sciences and Society, Aging Research Center, Karolinska Institutet and Stockholm University, Solna, Sweden; Department of Neurobiology, Care Sciences and Society, Aging Research Center, Karolinska Institutet and Stockholm University, Solna, Sweden; Department of Neurobiology, Care Sciences and Society, Aging Research Center, Karolinska Institutet and Stockholm University, Solna, Sweden; Stockholm Gerontology Research Center, Stockholm, Sweden; Department of Neurobiology, Care Sciences and Society, Aging Research Center, Karolinska Institutet and Stockholm University, Solna, Sweden; Stockholm Gerontology Research Center, Stockholm, Sweden; Department of Neurobiology, Care Sciences and Society, Aging Research Center, Karolinska Institutet and Stockholm University, Solna, Sweden; Department of Basic Psychology, Psychobiology and Methodology of Behavioural Sciences, University of Salamanca, Salamanca, Spain; Department of Neurobiology, Care Sciences and Society, Aging Research Center, Karolinska Institutet and Stockholm University, Solna, Sweden; Stockholm Gerontology Research Center, Stockholm, Sweden; Department of Neurobiology, Care Sciences and Society, Aging Research Center, Karolinska Institutet and Stockholm University, Solna, Sweden; (Medical Sciences Section)

**Keywords:** Gerontology, Health heterogeneity, Longevity, Physical function, Successful aging

## Abstract

**Background:**

Physical resilience (PR), the ability to recover from health adversities, is thought to buffer health challenges during aging. However, PR’s association with mortality and its ability to offset the negative effects of genetic susceptibility to shorter lifespan remains unknown.

**Methods:**

Data on 3 041 individuals (age: 60+) from the Swedish National Study on Aging and Care in Kungsholmen (SNAC-K) were analyzed. Physical resilience was assessed at baseline (2001–2004) using residual gait speed for a given level of chronic diseases, medications, and sociodemographics, categorized as low (residual *SD*’s ≤ −1), moderate (−1 < *SD* < 1), or high resilience (*SD* ≥ 1). A genetic risk score was derived from 4 single nucleotide polymorphisms linked to longevity (*hTERT, APOE, TOMM40, IGF-1R*). Cox proportional hazard models and Laplace regression examined 18-year mortality and median survival, respectively. Physical resilience was assessed as the moderator of the genetic risk score-mortality association in stratified analysis.

**Results:**

Compared to individuals with moderate PR, those with low resilience had higher mortality risk (HR: 1.28; 95% CI [1.09, 1.51]), with the opposite pattern in those with high PR (HR: 0.71; 95% CI [0.60, 0.84]). Above-median levels of genetic risk score were associated with increased mortality risk (HR: 1.34; 95% CI [1.18, 1.52]). Stratified by PR, mortality risk associated with higher genetic risk score was elevated among those with low and moderate resilience but not among older adults with high resilience.

**Conclusion:**

Physical resilience appears to partly modify mortality risk associated with genetic predisposition to shorter survival. Fostering PR could benefit personalized therapeutic strategies to support healthy aging.

Health during aging is characterized by considerable interindividual heterogeneity (1). The rate of functional decline varies not only among older adults with different diseases but also within individuals with the same type of illness (2). In line with the WHO model of healthy aging, whereby functional independence is emphasized over the absence of disease (3), physical resilience (PR) describes an individual’s ability to withstand decline or recover function in the face of age-related losses or diseases. Believed to be shaped by psychosocial factors, genetics, physical reserves, environmental factors, and life experiences PR could help explain vast aging health heterogeneities (4).

While the concept of PR is generally well accepted, there is no universally agreed-upon measure of PR or methodology for quantifying it. Previous studies have adopted different approaches to capture PR, including resilience rating scales (5,6), experimental tools such as fatigability tests (7,8), or detailed time series of physical functioning (8,9). Recently, a measurement strategy of PR that exploits the discrepancy between observed and expected functional status has been suggested (10–13). A residual approach identifies as resilient those older adults whose functional performance exceeds the levels predicted by their clinical and socioeconomic profiles (10).

Recent studies employing residual techniques to measure PR have varied in their choice of functional indicators from which to extract excess performance. One study focused on residual frailty (11), while 2 others considered physical function, 1 utilizing residuals in the Short Physical Performance Battery (SPPB) (13), and another in gait speed (GS) (12). Gait speed may be particularly suitable for deriving PR, as it is a complex function involving the integration and synergy of multiple physiological systems (14). Impaired GS (< 0.8 m/s) is widely regarded as a marker of dysregulation indicative of accelerated aging (15) and has been routinely linked with mortality (16). While GS generally tracks individual clinical status, with a mean difference of 0.24 m/s reported between older adults without a chronic condition and those with ≥ 3 chronic conditions (17), there is also considerable heterogeneity in the clinical disease–GS association (18). Physical resilience assessed as GS remaining after regressing out age, sex, clinical diseases, and medication was found to be associated with a reduced risk of falls in a study population of 70-year-olds from the United States (12). Whether PR as measured by residual GS is associated with mortality, a holistic measure of old-age health, is unknown.

Physical resilience’s recovery-enhancing potential has been largely scrutinized in relation to an acute event, for example, orthopedic surgery (19). In the absence of a well-characterized acute stressor, endogenous, genetically determined perturbations that progressively increase homeostatic disequilibrium could represent an alternative. Here, we consider genetic predisposition to shorter survival as such stressor. Longevity is characterized by a complex polygenetic phenotype, and several genes have been found to play a role in the heritability of survival (20). Implicated in protein instability (*APOE*) (21), mitochondrial dysfunction (*TOMM40*) (22,23), telomere shortening (*hTERT*) (24), and cellular senescence (*IGF-1R*) (25,26), these genetic variants are closely linked to the biological hallmarks of aging (27), and represent a source of homeostatic dysregulation, that gradually manifests as shorter survival (25,26,28–31). Whether PR can offset mortality risks associated with these genetic factors remains to be investigated.

The aim of this study is thus twofold: (a) to investigate the association between mortality and a measure of PR, based on the mismatch between GS, clinical disease- and socioeconomic profiles, and (b) to assess if the association between genetic predisposition to shorter survival and mortality is attenuated in the presence of high PR.

## Method

### Study Design and Participants

This study utilizes longitudinal data from the Swedish National Study on Aging and Care in Kungsholmen (SNAC-K). SNAC-K is an ongoing population-based study employing stratified random sampling to include cohorts aged 60, 66, 72, 78, and 81+ years at baseline examination, with an initial participation rate of 73%. Follow-up occurs every 6 years for younger cohorts (60–78 years) and every 3 years for older cohorts (age 78+ years) (32). The analytic sample size ranged from 2 971 to 1 944 in different models due to missing data on considered covariates (details on missing data are provided alongside each analysis conducted).

For this study, we analyzed longitudinal data from baseline (2001–2004) until wave 6 (2016–2019) of SNAC-K, resulting in a follow-up duration of up to 18 years. Surviving participants were censored at the end of the sixth follow-up (December 25, 2019). Of the 3 363 individuals who participated in the baseline examinations, we excluded 322 individuals with prevalent dementia (ascertained through a standardized procedure according to DSM-IV diagnostic criteria (33)), resulting in an eligible study population of 3 041 participants (Supplementary Figure 1).

SNAC-K was approved by the Karolinska Institutet Ethics Committee and the Regional Ethical Review Board in Stockholm. Written informed consent was obtained from all participants or from proxies in cases of cognitive impairment.

### Assessment of PR

We characterized PR based on residuals from a linear regression (12) in which baseline GS was the dependent variable and chronic disease burden, medication, sex, age (including its polynomials), and education were the independent variables. To assess GS, participants guided by a trained nurse were directed to walk at their usual pace at a distance of 2 × 6 m (including a turn) or 2.44 m, depending on whether they considered themselves to be fast or slow walkers (34). Studies have demonstrated that measuring GS over distances of 2.4 and 6 m produced comparable results (35).

Three resilience groups were defined based on the distribution of residuals. Those with large negative scores (residual *SD* ≤ −1), indicating that their observed GS was considerably slower than predicted by their clinical and sociodemographic characteristics, were classified as having *low resilience*; those with residual scores within 1 standard deviation (*SD*) of the mean, indicating good correspondence between observed and predicted GS were categorized as having *moderate resilience* and those with positive residual scores (*SD* ≥ 1), suggesting better-than-expected walking performance were classified as having *high resilience.*

### Assessment of Chronic Disease Burden and Medications

Chronic diseases comprised a total of 918 chronic diseases that were subsequently categorized into 60 disease groups (for details, see Supplementary Table 15) relevant to old age using a previously described and widely accepted approach (36). Chronic disease was defined as a condition that lasted over a longer period resulting either in persisting residual disability, decreased quality of life, or long-term care and treatment. The disease diagnosis of the SNAC-K participants was established through clinical examination, medical history, blood test results, and medication and was supplemented with data from hospital discharge registers. The diagnoses were aligned with the International Classification of Diseases, tenth revision (ICD-10) (36). The chronic disease burden was operationalized by summing the total number of chronic disease groups present in each individual at baseline (range 0–16).

As part of clinical assessment in SNAC-K, participants were asked to provide a list of their medications (both prescribed and over-the-counter) during the interview (36), and these were operationalized by summing the total number of medications for each individual at baseline (0–23).

### Assessment of Genetic Risk Score for Shorter Survival

In SNAC-K, a total of 103 single nucleotide polymorphisms (SNPs) were genotyped through MALDI-TOF analysis on the Sequenom MassARRAY™ platform during baseline and follow-up examinations. Among them, 4 SNPs (*hTERT* [rs2735940], *APOE* [rs7412, rs429358], *TOMM40* [rs2075650], *IGF-1R* [rs2229765]) were previously linked to survival and were used to generate a genetic risk score (Supplementary Table 1) (20,22,24,25,29,31,37–39). The selected SNPs have been identified in either genome-wide association studies, systematic reviews, or from at least 2 different original studies. To consolidate the APOE polymorphisms into 1 variable, we counted the number of ε4 alleles. The genotypes of the selected alleles were encoded according to the count of risk alleles: individuals with 2 risk alleles were labeled “2,” those with 1 risk allele as “1,” and individuals without any risk alleles were labeled “0.” The number of risk alleles was added up and subsequently categorized into “low” or “high” genetic risk using a median split. A similar approach to generating a genetic risk score has been recently described and used to estimate a metabolic genetic risk score (40).

### Outcome: Incidence of All-cause Mortality Over 18 Years

The date of death was retrieved from the Swedish Cause of Death Register that is linked to SNAC-K.

### Covariates

We considered sociodemographic, behavioral, and health-related factors as possible confounders of the association between PR and mortality. Sociodemographic variables comprise age (years), sex (women and men), education (elementary school [< 8 years], high school [8–12 years], and university [13+ years]), civil status (married, unmarried, divorced, in partnership [but living in separate households], widowed), and financial hardship in later life (ie, financial difficulties in the past year related to making payments [no/yes]). Behavioral factors included smoking status (never and ever), alcohol (no or occasionally, light- to moderate and heavy drinking), and physical activity according to the WHO recommendation for older adults (inactive, moderate, vigorous) (41). Health variables included functional impairments (sum of impairments in activities of daily living [ADL] and instrumental ADLs at baseline), depressive symptoms (Montgomery-Åsberg Depression Rating Scale [MADRS] at baseline), and body mass index.

### Statistical Analysis

We first derived resilience using the residuals from the model of GS as the dependent variable. Background characteristics of the sample across the levels of PR were presented descriptively. Cox proportional hazard (PH) models were used to assess mortality risks across PR groups. A similar analysis was performed with GRS as exposure to validate its association with mortality. The proportionality assumption in Cox models was assessed using Schoenfeld residuals. Alongside all Cox PH models, Laplace regression (42) was applied to examine differences in median survival time. The Hardy–Weinberg equilibrium was tested for the identified SNPs.

Lastly, an interaction analysis was performed to analyze the mortality effect of a combination of PR and genetic risk score. We also conducted stratified analysis whereby the effect of genetic risk score (high vs low) on mortality was estimated in sample subgroups defined by PR level (low, moderate, high). All analyses were stratified by age (< 78 years and ≥ 78 years), enabling the comparison of younger-olds and older-olds, who also varied in follow-up intervals (6 years for < 78 years and 3 years for ≥ 78 years).

### Sensitivity Analyses

We conducted a series of sensitivity analyses, including: (a) incorporating cognitive function into PR prediction models; (b) reclassifying resilience groups using other threshold levels; (c) exploring alternative physical function measures (Supplementary Table 13); and (d) respecifying chronic disease load using: (a) Charlson Comorbidity Index, (b) excluding obesity, osteoarthritis, and chronic obstructive pulmonary disease (COPD) from disease count; and (c) further removing risk factors (dyslipidemia and hypertension). Full details are described in the Supplementary Material (Supplementary Text 1 and 2). Additionally, we supply the baseline characteristics of participants included and excluded from the Cox regression analysis with GRS as main exposure due to missingness (Supplementary Table 14).

## Results

Descriptive analysis of the study population and by age group (< 78 years and ≥ 78 years) is presented in Table 1. The mean age of the sample was 73.24 (± 10.48), of which 62.94% were women.

**Table 1.** Baseline Characteristics of the Study Population According to Age-group*

	Full Sample *N* = 3 041	< 78 Years *N* = 1 758	≥ 78 Years *N* = 1 283
	Mean ± *SD* or *N* (%)	Mean ± *SD* or *N* (%)	Mean ± *SD* or *N* (%)
**Age** (years)	73.24 ± 10.48	65.43 ± 4.81	83.92 ± 5.56
**Sex**			
Female	1 914 (62.94)	1 009 (57.39)	905 (70.54)
Male	1 127 (37.06)	749 (42.61)	378 (29.46)
**Civil status**			
Married	1 373 (45.28)	987 (56.27)	386 (30.20)
Widowed	732 (24.14)	137 (7.81)	595 (46.56)
Unmarried	430 (14.18)	267 (15.22)	163 (12.75)
Divorced	421 (13.89)	296 (16.88)	125 (9.78)
In partnership	76 (2.51)	67 (3.82)	9 (0.70)
**Financial hardship in later life**			
No	2 602 (94.55)	1 574 (94.08)	1 028 (95.27)
Yes	150 (5.45)	99 (5.92)	51 (4.73)
**Education**			
Elementary	464 (15.31)	149 (8.49)	315 (24.69)
High School	1 511 (49.85)	803 (45.75)	708 (55.49)
University	1 056 (34.84)	803 (45.75)	253 (19.83)
**Smoking**			
Ever	446 (14.79)	329 (18.88)	117 (9.20)
Never	2569 (85.21)	1 414 (81.12)	1 155 (90.80)
**Alcohol**			
No or occasional	1 019 (33.79)	388 (22.21)	631 (49.72)
Light-to-moderate	1 498 (49.67)	1 000 (57.24)	498 (39.24)
Heavy drinking	499 (16.55)	359 (20.55)	140 (11.03)
**Physical activity**			
Inactive	872 (28.79)	355 (20.26)	517 (40.49)
Moderate	1 500 (49.52)	888 (50.68)	612 (47.92)
Vigorous	657 (21.69)	509 (29.05)	148 (11.59)
**Body mass index** (BMI, kg/m^2^)	25.67 ± 4.14	26.25 ± 4.14	24.81 ± 3.98
**Depressive symptoms (**MADRS score)	2.67 ± 3.95	2.20 ± 3.62	3.32 ± 4.29
**Number of chronic diseases**	3.87 ± 2.40	2.97 ± 1.89	5.12 ± 2.47
**Numbers of prescribed drugs**	3.81 ± 3.29	2.97 ± 2.98	4.96 ± 3.36
**Number of ADL + IADL**	0.51 ± 1.53	0.10 ± 0.64	1.09 ± 2.12
**Gait speed** (meters per second, m/s)	1.02 ± 0.44	1.22 ± 0.32	0.73 ± 0.41

*Notes*: ADL = activities of daily living; BMI = body mass index; IADL = instrumental ADL; MADRS = Montgomery-Åsberg Depression Rating Scale. Sum ADL and IADL.

^*^Missing data: smoking (*n* = 26); ADL + IADL (*n* = 118); BMI (*n* = 125); MADRS (*n* = 112); physical activity (*n* = 12); alcohol (*n* = 25); financial hardship (*n* = 289); civil status (*n* = 9); education (*n* = 10); gait speed (*n* = 49); numbers of prescribed drugs (*n* = 11).

### Derivation of PR

Supplementary Table 2 shows the linear regression model in which GS was the dependent variable, and number of chronic disease burden, number of medications, age (and its polynomials), sex, and education were the independent variables. All independent variables were statistically significant and explained nearly half of the total variance in GS (adjusted *R*^2^ = 0.47). Based on model residuals, 15% of the sample were classified as having low resilience (*SD* ≤ −1), 72% as moderate (−1 < *SD* < 1), and 13% as high (*SD* ≥ 1). Baseline characteristics of the study sample according to resilience category (low, moderate, high) are presented in Table 2. The correlation between PR and GRS was −0.01 (*p* = .671).

**Table 2.** Baseline Characteristics by Resilience Groups^*^^†^

	Low Resilience (*n* = 447)	Moderate Resilience (*n* = 2 144)	High Resilience (*n* = 380)	*p* Value
	Mean ± *SD* or %			
**Age** (years)	75.51 ± 10.52	71.97 ± 10.24	76.86 ± 10.07	<.001
**Sex**				.785
Female versus men	63.31	62.45	64.21	
**Civil status**				<.001
Married	37.44	47.59	44.74	
Widowed	31.84	20.88	31.58	
Unmarried	16.82	14.06	10.26	
Divorced	13.00	14.62	11.32	
In partnership	0.90	2.85	2.11	
**Financial hardship in later life**				<.05
Yes versus no	8.89	4.72	5.67	
**Education**				<.05
Elementary	17.23	14.09	18.42	
High School	52.13	49.11	52.37	
University	30.65	36.80	29.21	
**Smoking**				
Never versus ever	81.96	84.74	92.59	<.001
**Alcohol**				<.001
No or occasional	49.77	30.57	31.13	
Light-to-moderate	41.14	50.98	54.35	
Heavy drinking	9.09	18.45	14.51	
**Physical activity**				<.001
Inactive	58.39	24.04	16.84	
Moderate	33.56	52.38	53.68	
Vigorous	8.05	23.58	29.47	
**Body mass index** (BMI, kg/m^2^)	25.48 ± 5.16	25.81 ± 4.03	25.08 ± 3.25	<.05
**Depressive symptoms** (MADRS)	3.89 ± 5.31	2.46 ± 3.66	2.29 ± 3.29	<.001
**Number of chronic diseases**	4.32 ± 2.46	3.64 ± 2.36	4.61 ± 2.30	<.001
**Numbers of drugs**	4.12 ± 3.31	3.60 ± 3.29	4.51 ± 3.12	<.001
**Number of ADL + IADL**	1.33 ± 2.22	0.34 ± 1.17	0.19 ± 0.99	<.001
**Gait speed** (m/s)	0.44 ± 0.35	1.08 ± 0.34	1.39 ± 0.33	<.001
**Genetic risk score (GRS)**				.769
High versus low	52.20	52.71	50.48	

*Notes*: ADL = activities of daily living; IADL = instrumental ADL; MADRS = Montgomery-Åsberg Depression Rating Scale. Sum ADL and IADL.

^*^Resilience defined from gait speed residuals. Residuals are grouped according to a standard deviation split, labeled as low (**≤**−1 *SD*), moderate (− 1 < *x* < 1 *SD*), high resilience (**≥ **1 *SD*).

^†^Missing data by resilience group: low resilience: smoking (*n* = 9); ADL + IADL (*n* = 31); BMI (*n* = 45); MADRS (*n* = 16); alcohol (*n* = 7); fin. hard. (*n* = 76); GRS (*n* = 129); civil status (*n* = 1). Moderate resilience: smoking (*n* = 7); ADL + IADL (*n* = 53); BMI (*n* = 53); MADRS (*n* = 65); phys. act. (*n* = 2); alcohol (*n* = 8); fin. hard. (*n* = 151); GRS (*n* = 408); civil status (*n* = 3). High resilience: smoking (*n* = 2); ADL + IADL (*n* = 12); BMI (*n* = 6); MADRS (*n* = 12); alcohol (*n* = 1); fin. hard. (*n* = 27); GRS (*n* = 69).

### The Association Between PR and Mortality

Median survival time for mortality was 10.52 years (± 4.82) over a total period of 18.26 years. Relative to older adults with moderate PR, participants with low resilience had a significantly higher hazard of death (HR: 1.28; 95% CI [1.09, 1.51]) in a fully adjusted model (Table 3). Correspondingly, high resilience was associated with a reduced risk of mortality (HR: 0.71; 95% CI [0.60, 0.84]). Of note, in older adults below 78 years, it was the detrimental effect of low (vs moderate) resilience that was most pronounced (HR: 1.63; 95% CI [1.26, 2.09]), while in those above 78 years, the dominant effect was that of high (vs moderate) resilience (HR: 0.66; 95% CI [0.55, 0.89]).

**Table 3.** Survival Analysis With Physical Resilience as Main Exposure—Hazard Ratios and 95% Confidence Intervals, Alongside Laplace Regression Estimates

Resilience Groups	Minimally Adjusted^†^	Multivariate^‡^	
	Cox regression outcome: all-cause mortality	Cox regression outcome: all-cause mortality	Differences (95% CI) in median survival (years)
	***N* = 2** **815; no. of deaths = 1** **656**	***N* = 2** **361, no. of deaths = 1** **240**	
Low	1.64 (1.45, 1.85)^*^	1.28 (1.09, 1.51)^*^	−1.14 (−1.99, −0.30)^*^
Moderate	1.00	1.00	Reference
High	0.62 (0.53, 0.71)^*^	0.71 (0.60, 0.84)^*^	1.85 (1.11, 2.59)^*^
**<78 years**	**(*n* = 1** **584; no. of deaths = 539)**	**(*n* = 1** **431; no. of deaths = 468)**	
Low	2.18 (1.76, 2.70)^*^	1.63 (1.26, 2.09)^*^	−1.89 (−3.45, −0.33)^*^
Moderate	1.00	1.00	Reference
High	0.66 (0.47, 0.92)^*^	0.76 (0.53, 1.03)	1.05 (0.39, 2.07)^*^
**≥ 78 years**	**(*n* = 1** **233; no. of deaths = 1** **119)**	**(*n* = 930; no. of deaths = 832)**	
Low	1.44 (1.24, 1.68)^*^	0.99 (0.79, 1.23)	−0.24 (−1.10, 0.75)
Moderate	1.00	1.00	Reference
High	0.60 (0.51, 0.71)^*^	0.66 (0.55, 0.89)^*^	2.31 (0.97, 3.66)^*^

*Notes*: ADL = activities of daily living; IADL = instrumental activities of daily living; BMI = body mass index.

^*^ *p* < .05.

^†^Minimally adjusted: age and sex.

^‡^Multivariate: age, sex, education, ADL + IADL, depression, civil status, BMI, smoking, alcohol, financial hardship, physical activity.

Results from the multiadjusted Laplace models (Table 3) showed that compared to those with moderate resilience, older adults with low resilience had a median survival that was shortened by 1.14 years, whereas in those with high resilience, survival was prolonged by 1.85 years compared to moderate resilience.

### The Association Between GRS and Mortality

*hTERT* and *APOE* exhibited no significant departure from Hardy–Weinberg equilibrium. Despite a departure from the Hardy–Weinberg equilibrium, *IGF1-R* and *TOMM40* were still included in the risk score due to their relevance to the study objectives. High (above median) genetic risk was significantly associated with a higher hazard of death (HR: 1.34; 95% CI [1.18, 1.52]; reference: GRS below median). The point estimate was consistent across both age groups, albeit no longer statistically significant in the < 78 years group.

Laplace regression analysis showed decreased median survival in those with high versus low GRS (−0.80 years; 95% CI [−1.39, −0.21]). The direction of association in both multiadjusted Cox regression and Laplace regression was more pronounced among individuals aged 78+ years (Table 4). Harrell’s *C*-statistic (equivalent to area under the curve) in survival models indicating GRS’ discriminatory ability for mortality was 0.53.

**Table 4.** Cox Regression Analysis of All-cause Mortality With Genetic Risk Score of Shorter Survival as Main Exposure—Hazard Ratios and 95% Confidence Intervals, Alongside Laplace Regression Estimates

Genetic Risk Score^§^	Model 1^†^	Model 2^‡^	
	Cox regression analysis of all-cause mortality	Cox regression analysis of all-cause mortality	Differences (95% CI) in median survival (years)
	***N* = 2** **305; no. of deaths = 1** **301**	***N* = 1** **944; no. of deaths = 1** **027**	
Low	1.00	1.00	Reference
High	1.27 (1.14, 1.42)^*^	1.34 (1.18, 1.52)^*^	−0.80 (−1.39, −0.21)^*^
**< 78 years**	**(*n* = 1** **328; no. of deaths = 429)**	**(*n* = 1** **193; no. of deaths = 364)**	
Low	1.00	1.00	Reference
High	1.23 (1.02, 1.49)^*^	1.21 (0.98, 1.49)	−0.65 (−1.52, 0.22)
**≥ 78 years**	**(*n* = 977; no. of deaths = 872)**	**(*n* = 751; no. of deaths = 663)**	
Low	1.00	1.00	Reference
High	1.27 (1.11, 1.45)^*^	1.41 (1.20, 1.65)^*^	−1.16 (−2.19, −0.13)^*^

*Notes*: ADL = activities of daily living; IADL = instrumental activities of daily living; BMI = body mass index; GRS = genetic risk score; SNPs = single nucleotide polymorphisms.

^*^ *p* < .05.

^†^Model 1: GRS, age, and sex.

^‡^Model 2: GRS, resilience groups, age, sex, education, ADL + IADL, depression, civil status, BMI, smoking, alcohol, financial hardship, and physical activity.

^§^GRS computed based on a median split (high vs low) of total of risk alleles in the following SNPs: hTERT, IGF-1R, APOE4, and TOMM40.

### Interplay of PR and Genetic Predisposition for Mortality Risk

There was no statistically significant multiplicative interaction between PR and genetic predisposition to shorter survival. However, considering the high plausibility of a biological interplay, we opted to explore the interplay between PR and GRS in stratified analysis (Table 5). We found that the detrimental effect of high (vs low) genetic risk score was progressively reduced across resilience groups: it was highest in those with low PR (HR: 1.73; 95% CI [1.23, 2.44]), diminished in those with moderate PR (HR: 1.28; 95% CI [1.08, 1.49]), and no longer statistically significant in those with high PR (HR: 1.27; 95% CI [0.90, 1.79]). This interplay appeared most pronounced in older adults aged 78 years and above.

**Table 5.** Survival Analysis^†^ With Genetic Risk Score of Shorter Survival as Main Exposure Stratified by Physical Resilience Groups—Hazard Ratios and 95% Confidence Intervals

Genetic Risk Score^‡^	Low Resilience	Moderate Resilience	High Resilience
	(*n* = 230; no. of deaths = 163)	(*n* = 1 452; no. of deaths = 712)	(*n* = 262; no. of deaths = 152)
Low	1.00 (Reference)	1.00	1.00
High	1.73 (1.23, 2.44)^*^	1.28 (1.08, 1.49)^*^	1.27 (0.90, 1.79)
**< 78 years**	**(*n* = 125; no. of deaths = 63)**	**(*n* = 955; no. of deaths = 275)**	**(*n* = 113; no. of deaths = 26)**
Low	1.00 (Reference)	1.00	1.00
High	1.17 (0.66, 2.06)	1.05 (0.82, 1.34)	4.74 (1.21, 18.54)^*^
**≥ 78 years**	**(*n* = 105; no. of deaths = 100)**	**(*n* = 497; no. of deaths = 437)**	**(*n* = 149; no. of deaths = 126)**
Low	1.00 (Reference)	1.00	1.00
High	1.87 (1.19, 2.94)^*^	1.39 (1.14, 1.69)^*^	1.12 (0.77, 1.63)

*Notes*: ADL = activities of daily living; IADL = instrumental activities of daily living; BMI = body mass index; GRS = genetic risk score; SNPs = single nucleotide polymorphisms.

^*^ *p* < .05.

^†^Adjusted for age, sex, education, ADL + IADL, depression, civil status, BMI, smoking, alcohol, financial hardship, and physical activity.

^‡^GRS computed based on a median split (high vs low) of total of risk alleles in the following SNPs: hTERT, IGF-1R, APOE4, and TOMM40.

The minimum detectable HRs, given stratum size and number of deaths within it, were below the estimated HRs in low- and moderate-, but not in the high-PR group, suggesting that the lack of statistical significance may be due to loss of power.

The overall pattern of results remained consistent following a series of sensitivity analyses, detailed in the Supplementary Materials (Supplementary Tables 3–12).

## Discussion

In this study, we applied a residual approach to quantify PR based on the mismatch between GS and disease burden, medications, and sociodemographic factors. Our findings showed a gradient in mortality risk and median survival time across PR levels: relative to moderate resilience, individuals with low resilience had higher mortality and shorter survival, while the opposite was found for the high resilience group. In people with low resilience, high (vs low) GRS conferred a 73% higher mortality hazard. This association was attenuated considerably in those with moderate PR and was no longer statistically significant in those with high PR, although the loss of statistical power may account for the insignificant finding. Still, attenuating pattern underscores PR’s ability to mitigate some aspects of health decline, highlighting its value in explaining older adults’ health heterogeneities (43) and suggesting that it may play a significant role in healthy aging (4).

PR extends beyond traditional disease models and aligns with the WHO model of healthy aging, which emphasizes the ability to adapt to health stressors and maintain function over the mere absence of disease (3). Additionally, PR is related to concepts like reserve or homeostasis (44). Homeostasis refers to the body’s ability to maintain stable internal conditions ensuring equilibrium at the cellular or organ level, while reserve is thought to represent the buffering capacity to withstand stress or damage that is available within a specific system (4,44). In contrast, PR is believed to be a whole-person characteristic (4,44), designating the degree of which reserve capacity is utilized across multiple systems in response to health-related stressors (4). Given the inadequacy of “one size fits all” approaches in the face of routinely reported heterogeneities in health during aging (44), an improved understanding of PR could advance treatment plans and individualized therapeutic strategies aimed at preservation of functional abilities in late life (8).

Despite general agreement on PR at the conceptual level, a widely accepted methodology for quantifying it has yet to be established, leading to diverse measurement approaches in the literature (4). Some have examined recovery trajectories following elective stressors or fatigability tests, for example, monitoring heart rate during an exercise stress test (45) or measuring blood pressure changes during an orthostatic challenge (9,46). Applying these tests in older patients raises challenges, such as determining whether resilience in specific physiological subsystems reflects PR at a holistic level or whether responses in controlled conditions translate to resilience in real-world settings (8). Furthermore, a widely accepted PR scale that is both validated and generalizable has yet to be identified, and by focusing on psychological attributes related to recovery (5,6), existing scales may not fully align with the definition of PR, where reserve capacity and clinical phenotypes are relevant. A residual approach to PR measurement reconciles a frequent observation in clinical practice, whereby outcomes do not align proportionally with stressor burden, leading to clinical uncertainty (8). Importantly, by embracing the discontinuity between clinical diseases and functional outcomes, a residual approach can be applied across the spectrum of health adversities. For instance, an 80-year-old frail individual who regains more walking ability than expected after a stroke could also be classified as resilient (10). While our findings support this approach of operationalizing PR, we recognize that combining diverse tools might offer the most reliable method to capture such a multifaceted concept (10).

Similar to our results, previous studies employing the residual approach have demonstrated a link between higher PR and improved health (11,13). For instance, Wu and colleagues found that individuals with *lower frailty than expected* (ie, higher PR), experienced longer healthy lifespans, lower mortality, and reduced hospitalization rates. We argue that residual GS may offer a more nuanced metric for operationalizing PR than frailty. Gait speed is related to both physical and cognitive phenotypes (14), providing multisystemic insights into functioning and overall health. If PR represents the realization of physiological potential along a spectrum between homeostasis and frailty (8), frailty as a marker of systemic dysregulation (47) may lack precision in capturing less severe alterations mitigated or unmitigated through compensatory mechanisms inherent in PR. Notably, while GS is able to robustly discriminate across a continuum of clinical states (48), effectively bridging the body-mind connection (14), a considerable portion of variance in GS remains unexplained even after comprehensively accounting for both physical and brain parameters (49). This indicates that the determinants of GS are complex and suggests that factors linked to PR could play a role in both GS heterogeneity, as well as in its correspondence with clinical diseases, the latter leveraged as resilience in this study.

Our findings are largely consistent with 2 studies that utilized physical function residuals to characterize PR. One study, based on data from the National Health and Aging Trends (NHATS) study in the United States, considered SPPB variance after accounting for self-reported clinical diseases, age, and race, finding that greater SPPB residuals (indicating higher PR) were associated with lower risks of mortality, falls, and hospitalizations (13). Another study, using a smaller sample of U.S. adults aged 65 and older (*N* = 510), specifically examined GS residuals, and found protective associations between PR and fall risk (12). Our work advances these findings by (a) incorporating a broader set of clinically measured chronic diseases in deriving PR, rather than relying on self-reports; (b) using a substantially longer follow-up for mortality; and (c) examining the mitigating capacity of PR in relation to genetic predisposition to shorter survival. Notably, in sensitivity analyses, we examined other measures of physical function (extremity strength and balance) as alternatives for deriving PR residuals, finding that models using GS performed better in terms of fit and assumption compliance. Indeed, it has been shown that GS is a valuable singular indicator of physical function with minimal loss in discriminatory power compared to aggregate scores (50). While our findings highlight the positive association between PR (as GS residual) and survival, the precise mechanisms underlying this relationship remain unclear. These mechanisms may align with a so-called “black box paradigm” (51), suggesting that the holistic interconnectedness of factors beyond clinical diseases and sociodemographics is likely relevant. While we adjusted our models for factors related to both GS variability and mortality, including anthropometrics, health behaviors, depressive symptoms, and socioeconomic status, GS residuals (and hence, resilience) could still be influenced by differences in personality, social support, or informal care, which need further investigation.

The beneficial effect of resilience is assumed to involve 2 pathways: (a) enhanced ability to resist damage and (b) enhanced recovery from damage that already occurred (9,10). By showing that individuals who maintained their GS despite clinical diseases also had a reduced risk of mortality, we may show resistance. Although we lack acute stressors, our findings imply that PR may play a role in recovery from homeostatic dysregulation acquired due to genetic predisposition to shorter survival. Indeed, it has been suggested that long-lived individuals either possess rare genetic variants that select on survival, lack conventional risk alleles for common diseases, or present mechanisms through which such variants can be compensated (20). Moreover, previous research has shown that lifelong engagement in intellectually stimulating activities, indicative of enhanced cognitive resilience, was able to mitigate the risk of dementia attributable to APOE-ε4 activity (52), thus enabling better health trajectories even in at-risk subpopulations. Our findings are generally consistent with these earlier results since our GRS also included APOE genes.

### Strength and Limitations

A noteworthy aspect of our research is the use of a residual approach to define PR, allowing for intuitive measurement of a complex concept. Another strength is the large population-based study design with a high participation rate (73% at baseline) and repeated follow-up assessments. Notably, we were able to consider up to 60 different disease groups and an objectively measured indicator of physical functioning (GS) in deriving PR.

Limitations include potential misclassification of PR, as residual size is directly linked to the quality and quantity of inputs in the initial GS model. We did not consider disease severity, measures of subclinical biological alterations (eg, inflammation, prediabetes), or other functional indicators (eg, cognitive function) in deriving resilience; we envisioned PR as discontinuity between clinical diseases and physical function and our objective was not to maximize prediction of GS. Other, studies may consider a more exploratory approach to modeling resilience. We did not assess the role of acute stressors in our PR operationalization; thus, we cannot capture precise recovery trajectories (although we use genetic predisposition to shorter survival as a source of endogenous perturbation). Characterizing PR into 3 discrete groups—a common practice in previous literature—likely concealed some within-group heterogeneity relevant to mortality, although we adjusted our analyses for a number of pertinent covariates. Relatedly, stratified analysis by PR levels has not been sufficiently powered for all groups. While some chronic conditions may have a stronger impact on GS than others, an unweighted count of chronic conditions is less susceptible to outcome-specific dependence induced by weighting and arguably provides a more comprehensive account of disease burden than comorbidity indices. Notably, the pattern of findings remained unchanged in sensitivity analyses employing the Charlson Comorbidity Index. Finally, GRS was defined in accordance with data availability and may neither contain relevant risk alleles nor assign appropriate weights according to their respective influences on longevity. Genetic risk score’ discriminatory ability for mortality was low, although our intention was not to maximize mortality prediction, but to assess interplay of genetic factors and resilience. Indeed, the estimated heritability in life expectancy is between 9% and 25% (20,53). This suggests that heritability may not be exclusively influential in longevity. Finally, SNAC-K participants are generally wealthier and healthier than the average Swedish population, which may limit the generalizability of the results. Still, it is noteworthy that differences in PR and its mortality association were observed even within such a relatively homogeneous and well-off sample. Implementation of PR tools in clinical practice may be challenging due to the need to estimate an individual’s expected GS. However, a previous study demonstrated an efficient way of deriving geriatric health charts from several health metrics (54).

## Conclusion

Greater levels of PR, defined as excess GS in relation to clinical disease, medications, and sociodemographic profiles, were associated with longer survival. Physical resilience could be of relevance for explaining the heterogeneity in aging health and even possibly help mitigate health decline due to some genetic risk factors.

## Supplementary Material

glaf101_suppl_Supplementary_Materials
